# Structural differences and differential expression among rhabdomeric opsins reveal functional change after gene duplication in the bay scallop, *Argopecten irradians* (Pectinidae)

**DOI:** 10.1186/s12862-016-0823-9

**Published:** 2016-11-17

**Authors:** Anita J. Porath-Krause, Autum N. Pairett, Davide Faggionato, Bhagyashree S. Birla, Kannan Sankar, Jeanne M. Serb

**Affiliations:** 1 Department of Ecology, Evolution, and Organismal Biology, Iowa State University, Ames, 50011 IA USA; 2 Department of Genetics, Development, and Cell Biology, Iowa State University, Ames, 50011 IA USA; 3 Department of Biochemistry, Biophysics, and Molecular Biology, Iowa State University, Ames, 50011 IA USA; 4 Interdepartmental Graduate Program in Bioinformatics and Computational Biology, Iowa State University, Ames, 50011 IA USA

**Keywords:** Rhabdomeric photoreceptor, R-opsin, Gene duplication, Melanopsin, Rhodopsin, Vision

## Abstract

**Background:**

Opsins are the only class of proteins used for light perception in image-forming eyes. Gene duplication and subsequent functional divergence of opsins have played an important role in expanding photoreceptive capabilities of organisms by altering what wavelengths of light are absorbed by photoreceptors (spectral tuning). However, new opsin copies may also acquire novel function or subdivide ancestral functions through changes to temporal, spatial or the level of gene expression. Here, we test how opsin gene copies diversify in function and evolutionary fate by characterizing four rhabdomeric (G_q_-protein coupled) opsins in the scallop, *Argopecten irradians*, identified from tissue-specific transcriptomes.

**Results:**

Under a phylogenetic analysis, we recovered a pattern consistent with two rounds of duplication that generated the genetic diversity of scallop G_q_-opsins. We found strong support for differential expression of paralogous G_q_-opsins across ocular and extra-ocular photosensitive tissues, suggesting that scallop G_q_-opsins are used in different biological contexts due to molecular alternations outside and within the protein-coding regions. Finally, we used available protein models to predict which amino acid residues interact with the light-absorbing chromophore. Variation in these residues suggests that the four G_q_-opsin paralogs absorb different wavelengths of light.

**Conclusions:**

Our results uncover novel genetic and functional diversity in the light-sensing structures of the scallop, demonstrating the complicated nature of G_q_-opsin diversification after gene duplication. Our results highlight a change in the nearly ubiquitous shadow response in molluscs to a narrowed functional specificity for visual processes in the eyed scallop. Our findings provide a starting point to study how gene duplication may coincide with eye evolution, and more specifically, different ways neofunctionalization of G_q_-opsins may occur.

**Electronic supplementary material:**

The online version of this article (doi:10.1186/s12862-016-0823-9) contains supplementary material, which is available to authorized users.

## Background

Organisms detect environmental stimuli using an array of sensory receptors. Changes to the genetic basis of these sensory receptors has been shown to allow organisms to exploit new ecological niches [1] or alter signaling between conspecifics [2], which can affect individual fitness and, ultimately, have evolutionary consequences for the species. Duplication of the genes that code for the sensory receptor proteins is thought to play an important role in expanding the diversity of sensory systems by providing new genetic material for novel phenotypes [3–6]. If gene duplicates are retained, they can follow one of three evolutionary fates (first outlined by [7]; see also expanded models reviewed by [8–10]). First, if both paralogs have the exact same function or suite of functions, the existence of a second copy can increase production levels of encoded protein (“gene conservation” [11]). Under this scenario, the second copy provides functional redundancy that can buffer against neutral loss-of-function mutations over evolutionary time. However, more dramatic functional divergence may occur following the duplication event. In the second scenario, if the original gene managed a suite of functions, such as enzymatic activity and signal transduction, the duplicated copies could subdivide these tasks (“subfunctionalization” [12]). Subfunctionalization of paralogs may include changes in spatial or temporal expression patterns [13] and may release one gene copy from adaptive constraint (“escape from adaptive conflict” model [14]) so that both copies can be optimized for particular tasks [15]. Finally, one copy of the duplicated gene can acquire a novel function while the other copy retains the original, pre-duplication function (“neofunctionalization” [7]).

In photosensory systems, the ability of an animal to become sensitive to a broader range of wavelengths is most often mediated by an increase in the number of opsins [16–22]. Opsins encode a class of G-protein coupled receptors (GPCRs), proteins with seven alpha-helical domains that transverse the cell membrane (helix, H1-7) interspaced by loops that extend into the cytoplasm (cytoplasmic loops, CL1-3) and outside of the photoreceptive cell (extracellular loops, EC1-3). Opsins covalently bind a light-absorbing vitamin-A derived chromophore, such as 11-*cis*-retinal, using a lysine residue in H7. Together, the opsin protein and chromophore molecule form a photopigment sensitive to a specific portion of the light spectrum. Photopigments are often characterized by the wavelength at which the absorbance of light is the greatest (λ_max_). When 11-*cis* retinal absorbs a light photon, it isomerizes to an all-*trans* state. As a result, the opsin undergoes a conformational change and releases a complex of heterotrimeric guanine nucleotide-binding proteins (G-proteins), which are specific to that opsin (reviewed in [23]). The dissociated alpha-subunit of the G-protein activates the phototransduction cascade through second messenger molecules. Depending on the particular transduction pathway initiated by opsin, the photoreceptor cell may either hyperpolarize (e.g., G_t_-protein coupled opsins in ciliary cells) or depolarize (e.g., G_q_-protein coupled opsins in rhabdomeric cells) [24]. Opsin specificity to its G-protein partner is regulated by G-protein binding sites [25] and is associated with particular amino acid motifs in the fourth cytoplasmic loop [26]. Phylogenetically, opsins group into clades based, in part, by the G-protein partner and to a lesser extent by photoreceptor type (rhabdomeric versus ciliary cells) [27, 28].

Because a photopigment can only absorb a portion of the light spectrum, increasing the number and diversity of opsins through gene duplication and divergence allows an expansion of the photoresponse to new wavelengths of light. This may lead to color discrimination, if the photopigments have different light sensitivities. Under this neofunctionalization model, changes in the amino acid residues at positions that interact with the chromophore (e.g., “spectral tuning sites”) shift the wavelength at which absorbance is the greatest (λ_max_) of the duplicated visual pigment. Thus, the potential advantages for organisms with multiple and genetically diverse photopigments include extending the range of spectral perception, new functionality under different light conditions, generation of wavelength-specific behaviors, or providing the molecular substrate in the retina for color vision (reviewed in [29]). Any of these phenotypes may allow an animal to occupy new or more heterogeneous photic niches [30, 31].

While it is well-documented that duplicated opsin genes most often attain a new λ_max_ by neofunctionalization [32–40] it is less understood what other phenotypic outcomes may follow the duplication of opsin genes (but see [21]). Photoreceptors in invertebrates occur in multiple tissue types and in different life stages, and can function as both ocular and extra-ocular sensory receptors [41–46]. Thus, in invertebrates, neofunctionalization of opsins may include co-option between tissues, organs, or life stages after a gene duplication event. In order to distinguish among different evolutionary outcomes of opsin duplication and what effect gene duplication may have in the evolution of the photoreceptive cells and organs in a given system [47], it is necessary to first identify and then characterize the diversity of opsin proteins that are present.

Here, we assess the evolutionary history of G_q_-opsins in scallop to examine the role of gene duplication in producing extant diversity. The molecular basis of photoreception in the scallop is complex. The mirror-type eyes of scallops contain at least two different phototransduction systems based on opsins that presumably couple with G_o_- and G_q_-proteins [48]. Previously, we identified a duplication event of scallop G_q_-protein coupled opsins that occurred over 230 Mya [49]. Because gene copies with identical gene function are unlikely to be maintained in the genome unless the new duplicate is advantageous [50], the long-term retention of these opsin duplicates in the scallop lineage suggests a fitness cost if the copies are not maintained. For these duplicates to persist over evolutionary time, opsin copies must have diverged phenotypically under one or more of the evolutionary fate models described above. To test this hypothesis, we determined the evolutionary fates of these duplicated scallop opsins. We first captured the genetic diversity of G_q_-protein coupled opsin genes (herein *opnGq* for the gene or the coding region, and OPNGq for the protein) by generating transcriptomes of photosensitive tissues from adult animals and placed the genetic diversity of scallop G_q_-opsins into an evolutionary framework by employing a phylogenetic analysis. We next asked how might these scallop OPNGq proteins interact with a chromophore. To do so, we capitalized on the x-ray crystallography data from the squid OPNGq (“squid rhodopsin”) [51, 52] to model the tertiary structure of the scallop OPNGqs. Then, we examined if the protein characteristics of each paralog differ. As a first approximation to identify differences in λ_max_ among scallop G_q_-opsins, we leveraged existing computational models that estimate electrostatic interactions between the amino acids and the chromophore of squid OPNGq and applied them to the scallop data. Finally, we examined differences in gene expression of *opnGq* paralogs across both ocular and extra-ocular photoreceptive organs. From these lines of evidence, we show that scallop G_q_-opsin paralogs differ in 1) the biochemical properties of amino acid residues interacting with the chromophore; 2) expression levels of the gene; and 3) spatial expression of the gene among light-sensitive tissues in the adult organisms.

## Methods

### Transcriptome assembly and gene analyses

Thirty-six adult individuals of the bay scallop, *Argopecten irradians* (Pectinidae), were collected from the Gulf of Mexico near Sanibel, Florida during July, 2012. The adults were kept in recirculating saltwater tanks under a light regime of 13 h of light and 11 h of dark per 24-h cycle. To maximize the likelihood of capturing all G_q_-opsin transcripts expressed, we collected tissues under both light and dark treatments (nine hours of light vs. nine hours of dark), with the expectation that the highest level of opsin expression would occur nine hours after sunrise [53, 54]. The tissues from dark-treated scallops were dissected under red-light. All eyes from the left and right mantles were collected and pooled for each animal (~60 eyes/individual). Small sections of mantle tissue were sampled along the anterior-posterior axis from both left and right valves and pooled for each individual. A portion of adductor muscle equivalent in volume to the dissected eye tissue was collected from each individual. RNA was extracted from the three tissue types using the Ambion RiboPure RNA extraction kit (Life Technologies). RNA samples from the tissues of one light-treated and one dark-treated individual were sent to the Iowa State University DNA Facility for library creation and transcriptome sequencing on an Illumina HiSeq2000. Nearly 1.5 trillion 100 base pair (bp) paired-end reads were generated from six libraries: light/dark eyes, light/dark mantle, and light/dark adductor. A *de novo* assembly of a reference transcriptome from all six libraries was created in the Trinity sequence assembly and analysis pipeline [55] by first normalizing the raw reads to remove redundancy with the Trimmomatic script, then assembling the quality trimmed reads. This assembly resulted in 231,391 transcripts with a contig N50 of 2078 and an average contig length of 971 bp. The assembled transcriptome data was given the reference name of “AirradFL.” Opsin sequences from two other scallop species [56] were used as queries to identify Gq-opsin sequences in the AirradFL reference transcriptome using BLAST. Putative opsin sequences from the AirradFL reference transcriptomes were blasted back to the NCBI nonredundant (nr) database to further confirm the sequence identities. Gene and protein nomenclature follows the general guidelines in invertebrate model organisms (e.g., http://www.wormbase.org), where gene and transcript names (italicized) are composed of a three-letter species prefix, followed by a hyphen, the class (homolog) of the gene, and a number (e.g., *Air-opnGq1*). The number provides the order of gene discovery of paralogs within a species or lineage. Proteins use the gene name, with the gene abbreviation without italics and in all uppercase (e.g., Air-OPNGq1).

### Phylogenetic analysis

To determine the phylogenetic placement of putative scallop G_q_-opsins, we compiled G_q_-opsin sequences from genomes, transcriptomes or single genes from public databases at Genbank (http://www.ncbi.nlm.nih.gov/genbank/) and assembled data from Porter et al. [27] (Additional file 1: Table S2). We queried all five publically-available molluscan genomes for additional G_q_-opsins: pearl oyster, *Pinctada fucata* (June, 2013); Pacific oyster, *Crassostrea gigas* (June, 2013); freshwater snail, *Biomphalara glabrata* (June, 2013); owl limpet, *Lottia gigantea* (June, 2013); and sea hare, *Aplysia californica* (June, 2013). G_q_-opsin sequences were found by blasting scallop opsins against predicted gene models from each molluscan genome using tblastx and an E-value cutoff of 1e-3. When gene models were not available, the genome contigs/scaffolds were used. The putative G_q_-opsins identified through BLAST were then reciprocally blasted back to the NCBI nonredundant (nr) database and subjected to phylogenetic analyses with known metazoan G_q_-opsins to confirm their identity.

Amino acid sequences of the 96 opsins from 42 taxa, including four annelids, 38 arthropods, 21 molluscs, and six platyhelminthes, (Additional file 1: Table S2) were aligned using MAFFT v 7.017 [57] as implemented in Geneious (v5.6.7). (http://www.geneious.com). This dataset included opsins from the G_i_- and G_o_-opsin families to test the monophyly of the G_q_-opsin clade. The G_o_-opsin from *Argopecten irradians* was used to root the phylogeny. The aligned dataset was then manually trimmed to remove long C- and N-terminus sequences and remove a single large (>50 aa) gap around position 258 in the H6. The trimmed, aligned dataset contained 355 amino acids. The best-fit model of protein evolution for this dataset was determined using ProtTest [58], which found the LG + G + I + F model [59] to have the lowest Akaike Information Criteria score (AIC). A maximum likelihood (ML) phylogeny of the aligned dataset was constructed using Randomized Axelerated Maximum Likelihood (RAxML) v 8 [60]. Node support was calculated using 1000 rapid bootstrap replications as implemented in RAxML. Using the same model of protein evolution, we also analyzed the data under Bayesian inference using MrBayes v3.2.6 [61] on the XSEDE tool available through the CIPRES Science Gateway [62]. We used the Metropolis Coupled Markov Chain Monte Carlo method with one cold and three hot chains for 3.1 million generations with a burnin of 1000 for two independent runs. Convergence was determined when the potential scale reduction factor (PSRF) approached 1.

### PCR confirmation of scallop opsin transcripts

All opsin transcripts were confirmed to be single genes by PCR amplification of the complete coding region with UTR-specific primers from both cDNA and genomic DNA (Qiagen DNeasy Blood and Tissue kit) (Additional file 2: Table S1). PCR products were size-screened using agarose gel electrophoresis, bands of expected size were gel extracted (Qiagen Qiaquick Gel Extraction kit) and cloned using chemically competent *E. coli* cells (TOPO TA Cloning Kit with pCR2.1-TOPO). Positive colonies from blue-white screening were Sanger sequenced using an ABI 3730 Capillary Electrophoresis Genetic Analyzer at the Iowa State University DNA Sequencing Facility. The resulting sequences were translated and compared against contigs from the transcriptome. Using the same approach, we confirmed that a large contig sequence containing two G_q_-opsin transcripts (*Air-opnGq3* and *Air-opnGq4*) and an intergenic region of ~1690 bp was present in the genome. Because repetitive motifs can indicate gene duplication due to transposable elements [63], we searched for repetitive motifs in this intergenic region. To do so, the nucleotide sequence of the whole contig was screened with the RepeatMasker Web server v open-4.0.5 (http://www.repeatmasker.org/cgi-bin/WEBRepeatMasker) using the cross_match search engine on slow speed/sensitivity and the bivalves *Crassostrea gigas*, *Pinctada fucata*, and *Mizuhopecten yessoensis* as DNA sources.

### Homology modeling of scallop G_q_-opsins

To identify amino acid changes that may result in functional differences among scallop G_q_-opsins, we compare the Air-OPNGqs to the only molluscan opsin with a resolved crystal structure, the *Todarodes pacificus* “rhodopsin” (Tpa-OPSGq1; Genbank accession X70498) [51] We followed the amino acid numbering system of the squid where the first amino acid position in our alignment begins with the start codon (Met) of Tpa-OPNGq1. To examine the degree of resemblance among protein sequences, we calculated pairwise percent similarity of the scallop and squid amino acid sequences in the BLASTP 3.2.1 [64, 65] at NCBI (http://blast.ncbi.nlm.nih.gov/Blast.cgi?PAGE=Proteins).

We also used the protein alignment to identify amino acid residues that may interact with the chromophore. We applied a quantum mechanics/molecular mechanics model based on the crystal structure of Tpa-OPNGq1 [66], which predicts the involvement of 38 sites in spectral tuning of G_q_-opsins. We examined differences in the Air-OPNGq and Tpa-OPNGq1 sequences at these sites and noted changes in the biochemical properties of the residues.

Next we employed bioinformatic homology modeling to predict the tertiary structure of the four scallop G_q_-opsin proteins. These models were based on the template of the only available crystal structure for a G_q_-opsin, the rhodopsin from squid *Todarodes pacificus* 2ZIY [52]. The tertiary structure models of four scallop opsins (Air-OPNGq1, Air-OPNGq2, Air-OPNGq3 and Air-OPNGq4) were predicted using the Iterative Threading ASSEmbly Refinement (I-TASSER) server [67, 68]. The squid 2ZIY template was used to retrieve model proteins of similar folds from the Protein Data Bank (PDB) library using a locally installed meta-threading library. The continuous fragments excised from PDB templates were re-assembled into full-length models by replica- exchange Monte Carlo simulations and the unaligned regions were built by *ab-initio* modeling. The structure was then further refined with a second fragment assembly simulation. No restraints such as inter-residue contacts or inter-residue distances were specified for the modeling. For each G_q_-opsin, the top five predicted structures from I-TASSER were used for further quality assessment.

### Assessing the quality of the modeled tertiary structures

The quality of the modeled structures was assessed using the Ramachandran plot and the confidence score (C-score) (Additional file 3: Table S3) from the I-TASSER server. The Ramachandran plot is a graph of the backbone dihedral angles ψ against ϕ of the amino acid residues in the structure. Good quality models have more than 90% of the residues in allowed regions (i.e. most favored and additionally allowed regions) of the Ramachandran plot. The Ramachandran plot of the modeled structures was obtained using PROCHECK [69] which has been implemented as part of the PDBSum Server [70].

The C-score (from I-TASSER server) is a scoring function to rank models based on their quality and is defined using the significance of threading template alignments and the convergence parameters of the structure assembly simulations (for more details see [67]). C-scores are typically between −5 and 2 with higher values representing better models. However it has been observed that the C-score is particularly low (and negative) for membrane proteins. The “best” models of the four G_q_-opsin sequences were selected based on the highest C-score and maximum percentage of residues in the most favored and generously allowed regions according to the Ramachandran plots.

To quantify the overall shape differences among Gq-opsin tertiary structures, we performed a whole-molecule comparison between the predicted tertiary models calculating the Root-Mean-Square Deviation (RMSD) of the atomic positions of the alpha carbons between one opsin against each other. RMSD provided a quantitative computation of the average distance between the backbone atoms of two superimposed proteins. Variation in Air-OPNGq sequence length did not impact the RMSD values because a small portion of the N- and most of the C-termini were truncated from each sequence so the comparison occurs only between superimposed atoms. For RMSD comparison, only common one-to-one aligned residues, were included (V19 to K342). The values between each pair of structures were calculated using the standard ‘align’ program in PyMOL (The PyMOL Molecular Graphics System, Version 1.2r3pre, Schrödinger, LLC). Lower RMSD values indicate a higher similarity between structures.

### Scallop gene expression data

Paired-end RNA-seq data for three scallop tissues (eye, mantle, and adductor muscle) from the light treatment were aligned against the AirradFL assembly (nonredundant set of 231,391 transcripts grouped into 176,417 “genes”) using Bowtie v. 1.0.1 [71] followed by read abundance estimation with RSEM v. 1.2.9 [72] through the Trinity sequence and assembly pipeline v. 2013_2-25 [55]. Relative levels of expression in Fragments Per Kilobase per Million fragments mapped (FPKM) for a given transcript were calculated using the Trinity toolkit v. 2013_2-25 [55]. We accepted expression levels for a given transcript when the FPKM value was equal to or greater than one as a conservative approach to compare levels of relative expression among tissue types. Because tissues under the light treatment had the greatest levels of G_q_-opsin expression, only the results from light-treated tissues are reported here.

### Oyster gene expression data

To compare interspecific differences in G_q_-opsin expression patterns between bivalve taxa, opsin gene expression data for the Pacific oyster, *Crassostrea gigas*, were mined from the oyster genome database (OysterBase, http://www.oysterdb.com). We identified *opnGqs* from oyster by blasting our scallop G_q_-opsins against the database using the OysterBase blast tools with default settings. Gene expression data in RPKM (Reads Per Kilobase per Million) of the oyster G_q_-opsins (*Cgi-opnGqs*) were curated for each adult tissue type (digestive gland, gills, gonad, hemolymph, labial palp, mantle, and pallial mantle) and larval life stages (trochophore, D-shape larva, umbo larva, and pediveliger) from the website (OysterBase, http://www.oysterdb.com) and supplementary data tables (Table S12, S14) in Zhang [73]. However, comparing gene expression changes between the oyster (in RPKM) and scallop (in FPKM) tissues could only be described in relative terms.

## Results

### Transcriptomic and phylogenetic analyses reveal four G_q_-opsin genes in scallop

**Fig. 1:**
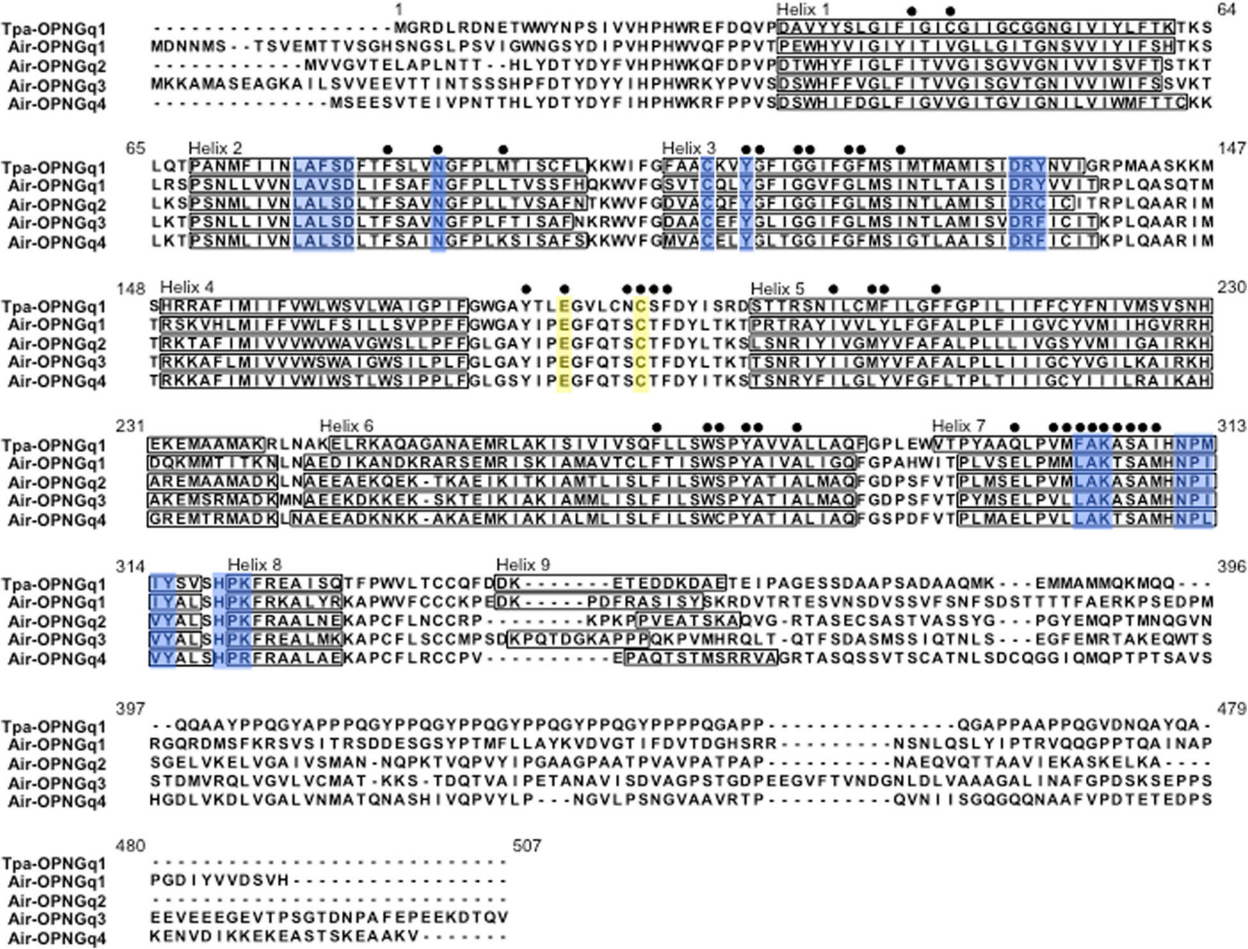
Amino acid alignment of G_q_-opsins from scallop (Air-OPNGq1-OPNGq4) and squid, *Todarodes pacificus* (Tpa-OPNGq1). The alpha-helix domains are based on protein structure homology modeling (this study) or have been adapted from Shimamura et al. [52]. Sequence motifs described in Table 2 are in *blue*; residues important for structural confirmation are in *yellow*. Numbering of amino acid positions begins with the start codon (Met) of Tpa-OPNGq1

To determine the number of G_q_-opsin genes in scallop, we performed deep transcriptome sequencing of tissue-specific libraries derived from dissected eyes, mantle tissue, and adductor muscle of *Argopecten irradians*. From light and dark treated animals, four transcripts were identified as putative *opnGqs* using a similarity-based analysis pipeline described in Pairett and Serb [56], which we named *Air-opnGq1*, *Air-opnGq2*, *Air-opnGq3*, and *Air-opnGq4* with ascending numbering according to the history of discovery (GenBank accession numbers KT426908, KT426909 KT426910, and KT426911). Visual inspection of the back mapped reads to each identified G_q_-opsin sequence did not show any obvious misassembled regions or mismatches. The proteins varied in amino acid percent similarity (the ratio of residues with similar physio-chemical properties shared between two sequences), which were the greatest between Air-OPNGq2 and Air-OPNSGq3 at 80.9%, and lowest between Air-OPNGq1 and Air-OPNGq4 (72.9%) (Table 1). Amino acid percent similarity was more conserved between the aligned Helix 1 (H1) through H7, and ranged from 92.6% (Air-OPNGq2 versus Air-OPNGq3) to 76.9% (Air-OPNGq1 versus Air-OPNGq4) (Table 1). Transcripts also differed in the sequence length from the first Met codon to the beginning of H1 (35–49 amino acids) and between the end of H7 and the stop codon (135–184 amino acids) (Fig. 1; Table 2).

**Table 1:** Percent similarity (below diagonal) and RMSD (above diagonal) of scallop (Air) and squid (Tpa) proteins

	Air-OPNGq1	Air-OPNGq2	Air-OPNGq3	Air-OPNGq4	Tpa-OPNGq1
Air-OPNGq1	-	0.378^b^	**0.354**	0.489	0.589
Air-OPNGq2	74.7 (78.9) ^a^	-	0.408	0.603	0.503
Air-OPNGq3	74.7 (77.9)	**80.9 (92.6)** ^**c**^	-	**0.699**	0.601
Air-OPNGq4	72.9 (76.9)	76.9 (85.6)	74.6 (88.1)	-	0.549
Tpa-OPNGq1	**71.0 (72.4)**	73.4 (73.8)	75.2 (75.8)	73.3 (74.7)	-

^a^ Percent similarity of amino acid sequence alignments from first methionine to stop codons; values in parentheses are percent identity from Helix 1 through Helix 7

^b^ Atomic values in angstroms, where the lower the RMSD value, the higher is the similarity between structures

^c^ Numbers in bold indicate minimum and maximum values

**Table 2:** Sequence and structural motifs in scallop (Air) and squid (Tpa) G_q_-opsins

Motifs	Air-OPNGq1 493 aa	Air-OPNGq2 456 aa	Air-OPNGq3 519 aa	Air-OPNGq4 481 aa	Tpa-OPNGq1 448 aa
LxxxD TMII (pos 76–80)	LAVSD	LALSD	LALSD	LALSD	LAFSD
Disulfide bond	C108, C186	C108, C186	C108, C186	C108, C186	C108, C186
Hydrogen bond with Schiff base	N87, Y111	N87, Y111	N87, Y111	N87, Y111	N87, Y111
E/DRY TMIII (pos 132–134)	DRY	DRC	DRF	DRF	DRY
Counterion	E180	E180	E180	E180	E180
LAK TMVII (pos 305–307)	LAK	LAK	LAK	LAK	FAK
NPxxY TMVII (pos 311–315)	NPIIY	NPIVY	NPIVY	NPLVY	NPMIY
G-protein binding (pos 319–321)	HPK	HPK	HPK	HPR	HPK

The amino acid numbering system follows the amino acid position (pos) of squid rhodopsin

**Fig. 2:**
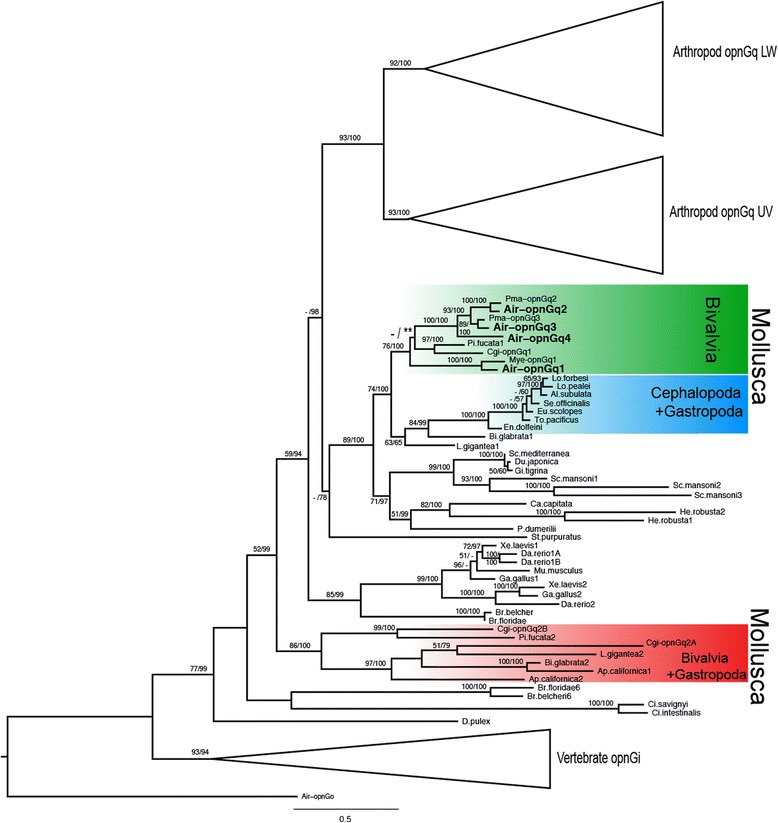
Maximum likelihood (ML) topology of G_q_-opsins. The phylogenetic tree is based on aligned amino acid sequences with scallop G_o_-opsin as the outgroup. Support values (>50%) of nodes were generated by 1000 bootstrap replicates in RAxML. Support values after the ‘/’ are posterior probabilities from a Bayesian phylogenetic analysis (BI). Support values <50% are indicated by a ‘-’. The single difference between the ML and BI topologies occurs within the bivalve G_q_-opsin clade (*green*) and is highlighted with an asterisk ‘*’. *Argopecten irradians* G_q_-opsins (Air-OPNGqs) from this study are in bold. Two molluscan G_q_-opsin clades were recovered, but only one clade (*green*) contained scallop G_q_-opsins from *Argopecten irradians* (Air-OPNGqs), *Mizuhopecten yessoensis* (Mye-OPNGqs), or *Placopecten magellanicus* (Pma-OPNGqs). Two large clades of arthropod opsins that represent UV and long-wavelength (LW) opsins and a vertebrate G_i_–opsin clade were collapsed for space

To determine how Air-OPNGqs were evolutionarily related to other G_q_-opsins, we conducted a phylogenetic analysis of their translated amino acid sequences with 96 metazoan opsins (Additional file 1: Table S2). Under both maximum likelihood and Bayesian inference, all four scallop sequences belonged to a clade that included G_q_-opsins from four other bivalve species: two oysters (*Pinctada fucata*, *Crassostrea gigas*) and two additional scallops (*Placopecten magellanicus*, *Mizuhopecten yessoensis*) (Fig. 2, green box). Within this clade, there was one difference between the ML and BI topologies, where ML placed the two oyster OPNGq1s as the sister group to the scallop G_q_-opsins 2–4, and the BI topology placed all bivalve OPNGq1s in a single clade (grey box in Additional file 4: Figure S1). However, values supporting these relationships were low (47% bootstrap support; 54 posterior probability). The bivalve-specific G_q_-opsin clade (OPNGq1-4) was the sister group to a clade of opsins from cephalopod and gastropod molluscs, and part of a larger clade of well-characterized vertebrate (e.g., melanopsin) and arthropod (e.g., *Drosophila* rhodopsin) G_q_-opsins (Fig. 2). A second molluscan G_q_-opsin clade was also recovered which contained oyster and gastropod opsins, but no scallop opsins (Fig. 2, red box). A complete, uncollapsed ML phylogram is available as a supplemental document (Additional file 5: Figure S2).

We then asked whether the four scallop G_q_-opsins possess the specific amino acid residues and sequence motifs required for photosensitivity. In addition to the seven transmembrane α-helices, it has been experimentally demonstrated that G_q_-opsin proteins require certain sequence motifs to maintain structural integrity and bind to the chromophore [74]. These include: 1) two Cys residues in the TM3 and EC2 domains that are involved in disulfide bond formation, 2) a Glu180 in the EC2 that functions as a counter ion to the positive charge of the protonated Schiff base [75], 3) a E/DRY motif near the TM3/CL2 boundary that helps stabilize the inactive-state conformation [76], 4) Asn87 and Tyr111 residues that are hydrogen binding partners for the protonated Schiff base [52], 5) a lysine residue in TM7 that is covalently linked to the chromophore, and 6) a conserved NPxxY motif in the TM7 [74]. We found that all four scallop proteins were invariant for the expected amino acid residues and motifs needed for correct conformation with the exception of the E/DRY motif (Table 2). This motif was variable among the scallop opsins, where Y134C in Air-OPNGq2 and Y134F in Air-OPNGq3 and Air-OPNGq4. In addition, we examined a motif (positions 319–321) in the fourth cytoplasmic loop, which has been experimentally demonstrated to be important for opsin-G_t_-protein interactions (positions 310–312 in bovine rhodopsin) [25]. Three of the four scallop opsins contain a HPK motif, an evolutionary conserved sequence that appears to be specific to G_q_-protein binding [77] (Table 2). Air-OPNGq4 had a HPR motif, but R has similar biochemical properties to K. Based on these data, we conclude that the four transcripts are indeed OPNGqs possessing the amino acid residues required for molecular stabilization, chromophore binding, and G-protein interaction and thus likely form photopigments.

### G_q_-opsin transcripts are not the result of alternative splicing

To determine whether the four different *opnGq* transcripts were the result of alternative splicing of the same gene, we developed target-specific primers (Additional file 2: Table S1) from the flanking UTR sequences for each *Air-opnGq*. We then compared these sequences derived from genomic DNA (gDNA) to transcripts derived from the transcriptomes. Alignments of 5′- and 3′-UTR DNA sequences and coding regions were identical between the transcripts and gDNA templates (data not shown). The flanking UTR sequences were not conserved and could not be unambiguously aligned across the four *Air-opnGqs* (Additional file 6: Figure S3).

While three of the four *Air-opnGq* sequences lacked introns, we identified a 393 bp intron within the region coding of H3 that was unique to *Air-opnGq1*. Additionally, gDNA sequencing determined that *Air-opnGq3* and *Air-opnGq4* were located in tandem, but in reverse orientation, with a 1690 bp intergenic region between the two coding regions. No repeat regions or putative transposable elements were identified in the intergenic region (data not shown). Variation in intron pattern and UTR sequences among the G_q_-opsins indicates that these four genes are most likely located on different physical places in the genome and are four separate loci.

### Predicted tertiary structure and chromophore-associated residues differ among scallop G_q_-opsins

We generated three-dimensional models for each Air-OPNGq using crystallography data from the squid “rhodopsin” [52] as a template for homology models. This allowed us to examine differences in the tertiary structure among the four G_q_-opsin sequences. The best model for each Air-OPNGq was selected based on the highest C-score and maximum percentage of residues in the most favored and generously allowed regions according to the Ramachandran plots (Additional file 3: Table S3). To quantify the overall shape differences among G_q_-opsin tertiary structures, we performed a whole-molecule comparison between the predicted tertiary models calculating the Root-Mean-Square Deviation (RMSD) of the atomic positions of the alpha carbons between one opsin against each other. Based on the RMSD of atomic values, tertiary structures differed from 0.354 to 0.699 Å, where lower RMSD values indicate higher similarity between structures (Table 1). Predicted tertiary structures were the most similar among Air-OPNGq1, Air-OPNGq2, and Air-OPNGq3 proteins (RMSD ranged between 0.354 and 0.408), while Air-OPNGq3 was most different from Air-OPNGq4 (RMSD = 0.699) (Table 1). Air-OPNGq3 and Air-OPNGq4 are more different in tertiary structure from each other than either are to squid rhodopsin (RMSD = 0.503 and 0.601).

**Fig. 3:**
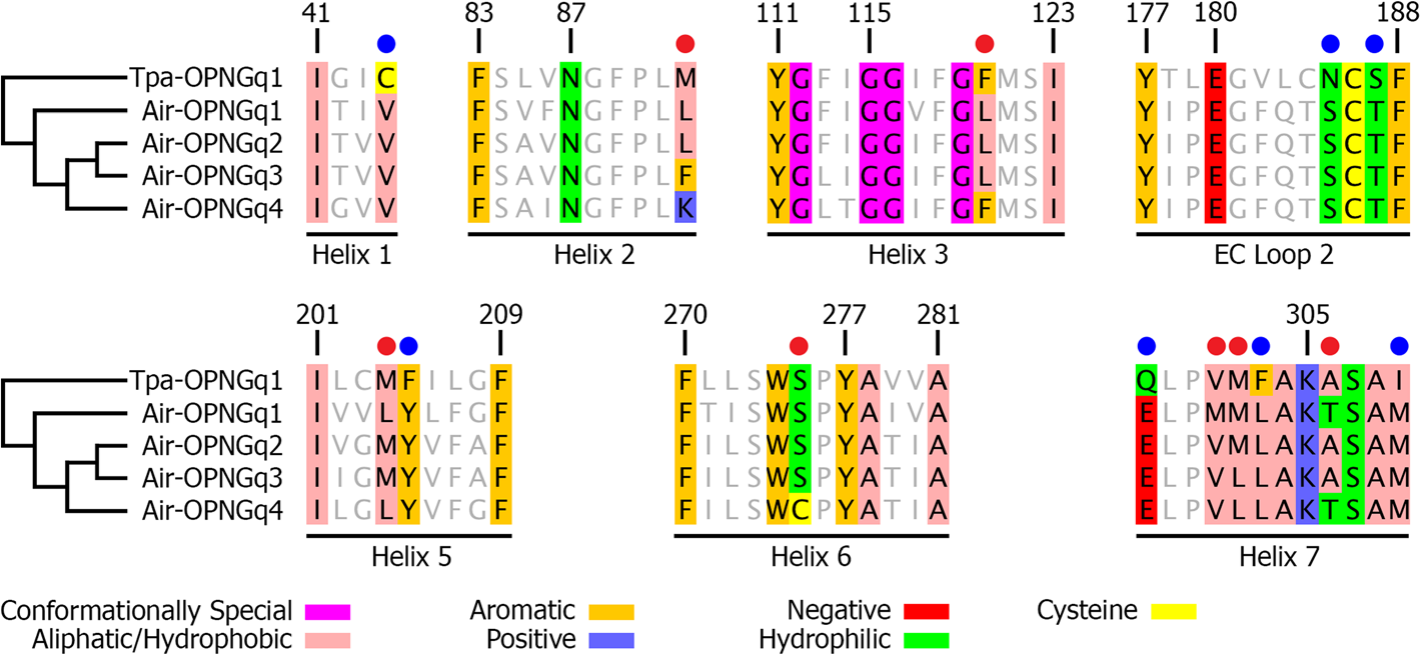
38 amino acid sites predicted to interact with chromophore in G_q_-opsins. Predicted amino acids forming the chromophore pocket from a QM/MM model based on the Tpa-OPNGq1 crystal structure from Sekharan et al. [66]. We have inferred the putative chromophore pocket in scallop Gq-opsins by aligning all Air-OPNGqs against Tpa-OPNGq1. *Blue dots* indicated seven amino acid positions where all scallop G_q_-opsins have the same residues and they differ from the Tpa-OPNGq1. *Red dots* identify the seven positions where amino acid residues differ among the four scallop G_q_-opsins. Numbering is based on Tpa-OPNGq1. The residues are colored according to their physicochemical properties under the zappo color scheme in Jalview v2 [105]. Numbering of amino acid positions begins with the start codon (Met) of Tpa-OPNGq1; EC, extra cellular loop

We then examined if the positions predicted to interact with the chromophore differ in their residues among the four scallop G_q_-opsins. We employed results from a quantum mechanics/molecular mechanics (QM/MM) model based on the Tpa-OPNGq1 crystal structure [66]. This model predicts 38 amino acid sites that may play a role in spectral tuning of G_q_-opsins. The scallop G_q_-opsins differed from the Tpa-OPNGq1 at seven of the 38 positions, but only three of these had residues with another biochemical property (Fig. 3, blue dots). Among the four scallop G_q_-opsins, seven of the 38 positions varied (Fig. 3, red dots). At four positions, at least one of the scallop opsins had an amino acid residue with a different biochemical property. Position 92 was the most divergent among Air-OPNGq proteins and included nonpolar aliphatic/hydrophobic (Air-OPNGq1 and Air-OPNGq2) and aromatic residues (Air-OPNGq3), while Air-OPNGq4 had a positive polar residue (Lys) at this position. At position 275, a conserved serine was substituted by cysteine in Air-OPNGq4, and at position 306, adjacent to the lysine forming the Schiff base, Air-OPNGq1 and Air-OPNGq4 have an hydrophilic residue instead of an hydrophobic/aliphatic residue (Fig. 3).

### G_q_-opsins are differentially expressed across the eye, mantle and adductor muscle tissues

To determine whether the expression patterns from the four G_q_-opsins in *A. irradians* differ spatially, we compared the relative expression level of each G_q_-opsin among the six tissue-specific transcriptomes from adult animals collected after a nine-hour light treatment or a nine-hour dark treatment. We found that spatial expression of the four G_q_-opsins was consistent in the light and dark adapted animals (data not shown); however, tissues under the light treatment had the greatest levels of G_q_-opsin expression and we only the report these results here.

**Fig. 4:**
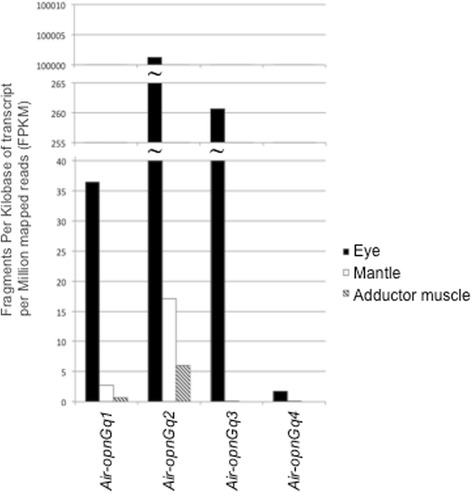
Expression profiles of scallop G_q_-opsin genes across three tissues from a single light-treated animal. Gene-specific mRNA levels were quantified using RNA-seq of tissue-specific libraries: eye (*black*), mantle (*white*), and adductor muscle (*striped*). Expression levels are reported in Fragments Per Kilobase of transcript per Million mapped reads (FPKM)

We found all four scallop G_q_-opsins were expressed in the eye. Outside of the eye, both *Air-opnGq1* and *Air-opnGq2* were expressed in the mantle, but only *Air-opnGq2* was expressed in the adductor muscle at levels above our expression threshold (≥1.0 FPKM; Fig. 4). As a general pattern across all tissue types, *Air-opnGq2* had the highest expression levels, while *Air-opnGq4* was expressed at the lowest level or not at all. When comparing relative expression levels in the eye, *Air-opnGq2* and *Air-opnGq3* had the highest relative expression levels with *Air-opnGq2* expression (10,001.27 FPKM) at ~38 times higher than *Air-opnGq3* (260.64 FPKM), 275-times higher compared to *Air-opnGq1* (36.46 FPKM), and over 5800-times higher *Air-opnGq4* (1.72 FPKM) (Fig. 4).

We then examined relative levels of gene expression in the Pacific oyster (*Crassostrea gigas*). Since this species is eyeless as an adult, we anticipated that its genome would contain a limited number of G_q_-opsins. However, our analyses identified three different G_q_-opsins in the *C. gigas* genome (*Cgi-opnGq1, Cgi-opnGq2A,* and *Cgi-opnGq2B*) that showed a degree of differential expression across tissues and life stages. *Cgi-opnGq1,* the oyster G_q_-opsin most closely related to the scallop opsins identified here (Fig. 2, green box), was found to have low (<1.0 RPKM) expression levels across the adult oyster tissues, but relatively higher expression in the larval umbo (2.508 RPKM) and pediveliger (21.355 RPKM) stages. *Cgi-opnGq2A* and *Cgi-opnGq2B* belonged to a second clade of gastropod and bivalve G_q_-opsins (Fig. 2, red box). *Cgi-opnGq2A* was most highly expressed in the adult tissues, with the labial palp (organs that move food to the mouth for ingestion) and pallial mantle (the tissue most similar to the scallop eye-containing mantle edge) showing the greatest *Cgi-opnGq2A* expression (2.290 RPKM and 4.080 RPKM, respectively). *Cgi-opnGq2B* showed the lowest expression across all tissues and life stages (<1.0 RPKM).

## Discussion

**Fig. 5:**
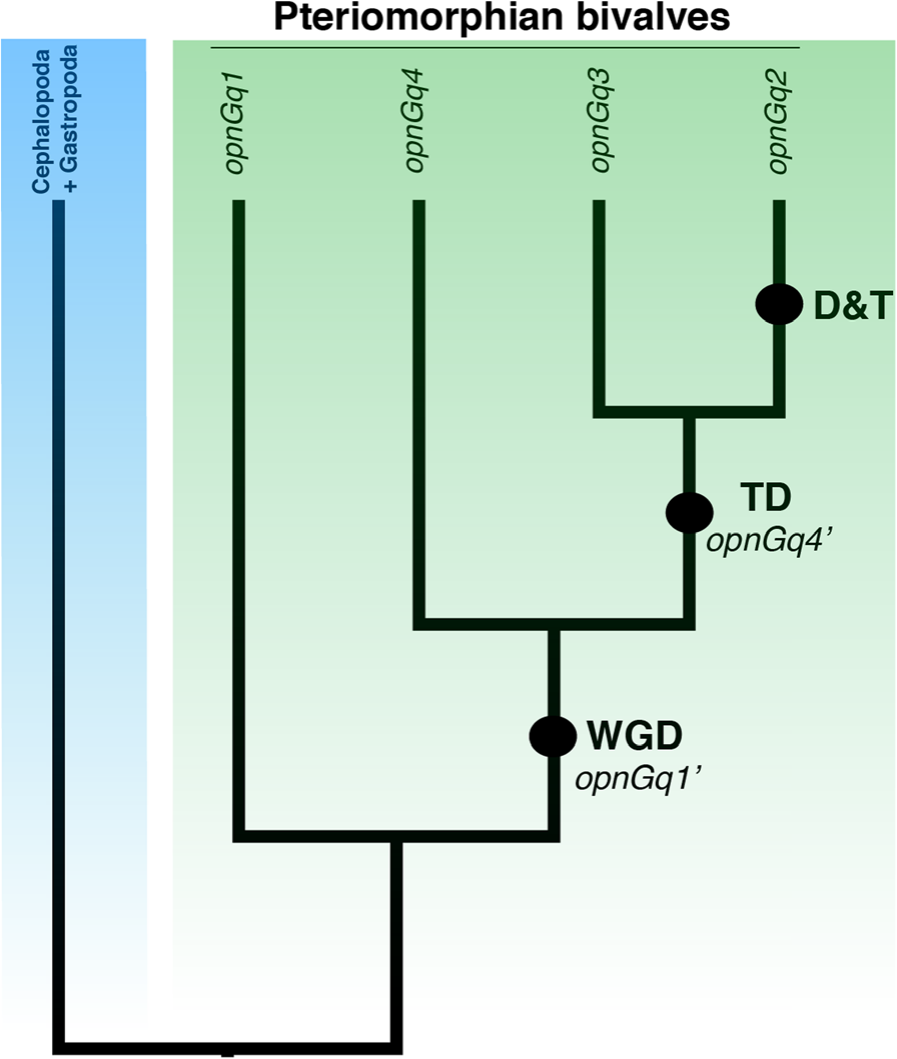
An evolutionary hypothesis describing G_q_-opsin duplications in Pectinioidea. Circles represent duplication events. At least five bivalve families (Ostreidae, Pteriidae, Pectinidae, Spondylidae, Limidae) in three Pteriomorphia orders (Ostreida, Pterioida, Pectinoida [49]) possess a G_q_-opsin gene homologous to *opn-Gq1*. One possible scenario is a whole genome duplication (WGD) event unique to the Pectinoida lineage [82] generated a second G_q_-opsin copy (*opn-Gq1’* to *opn-Gq4*). *Opn-Gq4* under went a tandem duplication (TD), *opn-Gq4’*. A final round of duplication generated *opn-Gq3* and *opn-Gq2.* The *opn-Gq2* copy was subsequently translocated (D&T) to another chromosome. While taxonomic sampling is not dense enough to determine whether these duplications are only in the family Pectinidae or in the order Pectinoida, current evidence supports the latter scenario as the Spondylidae and Limidae possess *opn-Gq2* homologs [49]. The other opsin paralogs (*opn-Gq3*, *opn-Gq4*) are either unsampled or may have been lost from these two families

The duplication of opsin genes is considered to be an important mechanism for the expansion of light-sensing capabilities of photosensory systems by either enhancing wavelength discrimination or increasing the spatial expression. While some of the best studied examples of photosensitivity expansion are the separate origins of color vision in insects [22, 42, 78] and vertebrates [17, 79, 80], where shifts in absorbance spectra are attributed to nonsynonymous substitutions to the coding region of one opsin copy, post-duplication fates of opsins need not be limited to changes in the coding region. Functional divergence of opsin copies can also be driven by changes to the untranslated regions of the gene, which contain regulatory elements influencing gene expression and translation. This latter phenomenon has been less studied in post-duplicated opsins (but see [81]). While we did not directly investigate regulation of scallop G_q_-opsin, our discovery of tissue-specific expression of G_q_-opsin paralogs in the scallop, *Argopecten irradians*, not only provides circumstantial evidence that there may be differences in regulatory regions, but offers an opportunity to investigate how these gene copies diversified in function and evolutionary fates. One-to-one matches between transcript and genomic amplicons strongly support the presence of at least four G_q_-opsin paralogs in the *A. irradians* genome. All four genes were identified as G_q_-opsins by both sequence similarity and phylogenetic analysis, and are most likely the result of duplication events in a lineage that includes the orders Pectinoida and Limoida [49], either through whole genome duplication events [82] or duplication of small segments of the genome [83]. The specific timing of these events will require denser taxonomic sampling within the subclass Pteriomorphia, but if the phylogenetic pattern from our study holds, it would appear that *opnGq1* and *opnGq4* are derived from the first round of gene or genome duplication. Subsequently, *opnGq4* may have undergone a tandem duplication, and the paralog underwent a second round of duplication to create *opnGq2* and *opnGq3* (Fig. 5).

We present evidence that all four *Air-opnGqs* products, when reconstituted with the proper chromophore, could form photopigments. Each scallop Gq-opsin has the sequence motifs necessary for protein conformation and chromophore binding (Table 2). Tertiary structural models developed for each Air-OPNGq contain the expected protein domains and loops for a functional opsin protein. Interestingly, all four scallop protein models predict eighth and ninth cytoplasmic α-helices (Fig. 1), features unique to G_q_-opsins [51]. In the Tpa-OPNGq1 crystal structure, the C-terminus of H9 interacts with the cytoplasmic extension of H6, that together with H5 form a rigid column projecting 25 Å from the membrane surface; however the rotational freedom of H9 is restricted by its interactions with H8. Thus, others have predicted that this four-domain cytoplasmic feature, in conjunction with the HKP motif in H8 [26], functions as the recognition mechanism for specific G-protein partners [51]. In summary, our bioinformatic analyses support that all four scallop G_q_-opsins form photopigments that could be used to detect light. How might these gene copies have diverged after the duplication event? Molecular changes in paralogous scallop opsin genes appeared to have occurred both outside and within the protein-coding region.

We find differential gene expression across ocular and extra-ocular structures in the adult, suggesting there have been changes in the regulatory regions of scallop G_q_-opsin paralogs. Specifically, while all *Air-opnGqs* are expressed in eyes, the level of expression is vastly different (ranging from a 38- to 5815-fold difference). In addition, only two of the four G_q_-opsins, *Air-opnGq1* and *Air-opnGq2*, are significantly expressed outside of the eye, and presumably they are used in a nonvisual context such as the “shadow response” [84]. Taken together, these data suggest that scallop opsin paralogs are used in different biological contexts. Some may preferentially be employed in eyes (*Air-opnGq3* and *Air-opnGq4*), while others (*Air-opnGq1* and *Air-opnGq2*) are used for both ocular and extra-ocular based functions.

Spatial patterning and expression level differences among the scallop G_q_-opsin paralogs suggest they have undergone neofunctionalization since duplication. When we compare the scallop opsin expression data to the closest related bivalve with a sequenced genome, the Pacific oyster, *Crassostrea gigas* [73] we find a dramatic difference in the relative levels of gene expression and spatial patterning. From the oyster genome, we identified three G_q_-opsins, but only one (*Cgi-opnGq1*) was phylogenetically similar to the scallop opsins (Fig. 2). This *Cgi-opnGq1* is broadly expressed at low levels across the adult non-ocular tissues (e.g., 0.10 RPKM in mantle tissue to 0.29 RPKM in gonad) [73]. In contrast, the adult scallop has high levels of expression (up to 10,001.27 FPKM) of different G_q_-opsin gene copies in eyes, and low or no expression of these opsins in non-ocular tissues (Fig. 4). Could an increase in opsin expression level and/or greater number of gene copies be related to the origin of eyes? Currently available opsin sequences from bivalve species represent a very restricted taxonomic sampling. But based on the nearly ubiquitous shadow response in Bivalvia and Gastropoda, the few instances of eyes in bivalves [85], and the results from our study, we anticipate that the ancestral state for G_q_-opsin spatial expression in bivalves is across multiple tissue types while the derived condition of spatial expression is narrowed (limited) to eyes and may indicate functional specificity for visual processes. If one or both of the scallop opsin duplication events were concurrent with the origin of eyes, it would support the notion of neofunctionalization of the new G_q_-opsin copies.

Do the differential levels of gene expression indicate an even finer spatial partitioning of *Air-opnGqs*? We anticipate this to be the case. Depending on the scallop species, an adult animal can have between 35 to over 200 eyes along the mantle margins lining both valves (Serb, unpublished) that can vary in size [86, 87]. Visual fields from adjacent eyes overlap such that, as a conservative estimate, at least five eyes would convey similar information from a given point in the environment (estimated from a 30-eyed animal [88]). One way to reduce functional redundancy would be to distribute Air-OPNGq proteins of dissimilar absorbance spectra across non-adjacent eyes. However, due to the limitations of library construction, which required the pooling of all 60 eyes from one light- and 60 eyes from one dark-treated animal, we are unable to determine if a single eye expresses all or a just subset of *Air-opnGqs*. Furthermore, the expression pattern of *Air-opnGqs* at the level of single photoreceptors also needs to be elucidated. Since *Air-opnGqs* are phylogenetically similar to the first reported scallop G_q_-opsin in *Mizuhopecten yessoensis*, which is presumed to be co-expressed with G_q_-protein in rhabdomeric photoreceptors of the proximal retina (“depolarizing layer”) [48], we can predict that Air-OPNGqs will share a similar gross expression pattern. At a cellular level, it has been shown that more than one G_q_-opsin can be expressed in a single photoreceptor cell [89–92] and this can lead to a broader spectral range for a given photoreceptor if opsins differ in λ_max_ values. Thus, to understand how spatial partitioning may have changed as gene copies diversified phenotypically in the scallop, future work will require the development of probes specific to each *Air-opnGq* gene or protein.

Spectral sensitivity may differ among the scallop G_q_-opsin photopigments. We identified changes in amino acid sequence at seven sites that are predicted to influence spectral tuning of G_q_-opsins [66]. The electrostatic contribution of individual residues at these sites has been modeled previously on Tpa-OPNGq1 [66, 75]. Among the scallop G_q_-opsins, residues at position 92 had the most dissimilar biochemical properties (nonpolar aliphatic/hydrophobic in Air-OPNGq1 and Air-OPNGq2; aromatic in Air-OPNG3; positive polar in Air-OPNGq4). Position 306 is also of interest because there is a difference in charge and a presence/absence of a hydroxyl group. Air-OPNGq1 and Air-OPNGq4 have a polar, hydroxyl-bearing Thr306 while Air-OPNGq2 and Air-OPNGq3 contain a non-polar Ala306. Evidence from previous studies [93–95] suggests that shifts in λ_max_ values can be achieved via a change of charge (polar vs non-polar) or a gain/loss of a hydroxyl group that ultimately affects the electrostatic potential around the protonated Schiff base [66]. Based on our results, we hypothesize that the λ_max_ may differ among some or all of the Air-OPNGqs. This hypothesis contradicts results from previous studies where only a single λ_max_ value was measured for depolarizing rhabdomeric photoreceptors [96, 97]. While some of the earliest work on spectral sensitivity of scallops was based on behavior trials, and was unable to test specific visual pigments, photoreceptor cells, or account for extra-ocular photoreception (e.g., [98]), more sophisticated methods have been employed to record membrane potential changes of individual photoreceptor cells (e.g., [97, 99, 100]). Most recently, microspectrophotometry has been used on dark-adapted scallop retinas to measure λ_max_ directly [96]. For rhabdomeric photoreceptors of *A. irradians*, both intracellular recordings [97] and microspectrophotometry results [96] recover a single spectral curve with a λ_max_ value of ~500 nm. Though, with the limited number of photoreceptor cells examined (N = 4 versus N = 21 [96, 97]) and a 38- to 5815-fold higher expression level difference of *Air-opnGq2* to other *Air-opnGqs* (this study), it is unlikely that all four G_q_-opsins were sampled. An alternative approach will be needed to determine if there are any differences in λ_max_ by targeting individual Air-OPNGqs. One approach would be to directly test λ_max_ of each Air-OPNGq photopigment *in vitro*, but the well-known technical challenges of expressing G_q_-opsin proteins in transient heterologous systems will need to be overcome [101, 102] or stable transfection of cell lines [103] or animals [104] will need to be employed.

## Conclusions

Gene duplication and subsequent functional divergence of opsins have played an important role in expanding photoreceptive capabilities of organisms by altering what wavelengths of light are preferentially absorbed by photoreceptors (spectral tuning). However, new opsin copies may also acquire new or subdivide ancestral functions through changes to temporal, spatial or the level of gene expression. As the first molecular characterization of scallop G_q_-opsins, our study highlights how opsin duplication and diversification may not only affect the evolution of the visual system, but also non-visual photoreception. Sequence variation among the scallop G_q_-opsins suggests different biochemical properties of the proteins, which may translate into differences in light absorption and/or G protein affinity. Changes to spatial pattern and level of gene expression are illustrative of transitions between broad non-visual photoreception and eye-specific expression indicating neofunctionalization after opsin-duplication.

It is important to extend the taxonomic sampling of intraspecific opsin diversity in non-arthropod invertebrates in the future to understand diversification and plasticity of G_q_-opsins. As such, molluscs are a rich system to study protein evolution, but have been underused due to a lack of basic information about their genic composition. Our work demonstrates the need for more studies looking at the visual evolution of molluscs to further their impact on the fields of molecular, sensory, and evolutionary biology.

## Additional files

Additional file 1: Table S2. G_q_-opsin sequences included in the phylogenetic analysis. Asterisks represent sequences obtained through Porter et al. [27]. For additional information regarding sequence acquisition not available on Genbank, see supplementary material in Porter et al. [27]. (DOCX 25 kb)

Additional file 2: Table S1. Primers used to amplify scallop G_q_-opsins and intergenic region between *Air-opnGq3* and *Air-opnGq4*. (DOCX 13 kb)

Additional file 3: Table S3. Ramachandran plot values and C-scores for top G_q_-opsin models. For each Air-OPNGq, the top five models reported by I-TASSER were analyzed for their quality using PROCHECK and the C-score. All the reported models have > 90% of their residues in allowed regions of the Ramachandran plot, indicating a good quality model. The C-scores for the best models was in the range of −3 to −2. While these values are lower than the suggested cutoff of −1.5, this is not unexpected for GPCRs because there are relatively few solved GPCR protein structures and GPCRs often show high sequence diversity. The best model for each Air-OPNGq (highlighted) was selected as the structure having the highest C-score and highest percentage of residues in allowed regions of the Ramachandran plot. (DOCX 14 kb)

Additional file 4: Figure S1. Bayesian inference phylogram of G_q_-opsins. The phylogenetic tree is based on 96 aligned amino acid sequences with scallop *Argopecten irradians* G_o_-opsin as the outgroup. Support values at nodes are posterior probabilities >0.50. The grey box highlights a clade of bivalve *opnGq1* not recovered in the ML analysis. A black bar indicates the monophyletic G_q_-opsin clade. (DOCX 94 kb)

Additional file 5: Figure S2. Maximum likelihood phylogram of G_q_-opsins. The phylogenetic tree is based on 96 aligned amino acid sequences with scallop, *Argopecten irradians*, G_o_-opsin as the outgroup. Support values (>50%) of nodes were generated by 1000 bootstrap replicates in RAxML. A black bar indicates the G_q_-opsin clade. (DOCX 98 kb)

Additional file 6: Figure S3. Fifty base pair alignment of 5′- and 3′-UTRs from the four scallop G_q_-opsins. Vertical lines represent the beginning and end of the coding region. (DOCX 49 kb)
