# Supplementary material for: Structural differences and differential expression among rhabdomeric opsins reveal functional change after gene duplication in the bay scallop, Argopecten irradians (Pectinidae)
Source: BMC Evol Biol. 2016 Nov 17;16:250. doi: 10.1186/s12862-016-0823-9 (PMC5114761; doi:10.1186/s12862-016-0823-9)
Supplement: Supplementary file 1 — Gq-opsin sequences included in the phylogenetic analysis. Asterisks represent sequences obtained through Porter et al. [27]. For additional information regarding sequence acquisition not available on Genbank, see supplementary material in Porter et al. [27]. (DOCX 25 kb) [file 12862_2016_823_MOESM2_ESM.docx]

**Additional file 2: Table S1.** Primers used to amplify scallop G_q_-opsins and intergenic region between *Air-opnGq3* and *Air-opnGq4*.

| Gene/fragment | Primer Name | Sequence (5’-3’) | Material | Product Length (nt) |
| --- | --- | --- | --- | --- |
| *Air-opnGq1* | 212F | CGCCTTATCACTCCGCAC | cDNA | 1630 |
|  | 1841R | CCTCAGTCATATCCAAGATGCC |  |  |
| *Air-opnGq1* | 212F | CGCCTTATCACTCCGCAC | gDNA | 1009 |
|  | 1100R | GATGGCTGAGAGCATATACCACTGG |  |  |
| *Air-opnGq2* | 38F | GCTACCACCCAGGGTACTCC | cDNA | 1585 |
|  | 1552R | CAGCAAACACGTGACTTCCATC |  |  |
| *Air-opnGq2* | 38F | GCTACCACCCAGGGTACTCC | gDNA | 1585 |
|  | 1552R | CAGCAAACACGTGACTTCCATC |  |  |
| *Air-opnGq3* | 807F | CCGTTGGGGTTTATCATCTC | cDNA | 1741 |
|  | 2548R | GACAAACTGGAAATGAACTC |  |  |
| *Air-opnGq3* | 807F | CCGTTGGGGTTTATCATCTC | gDNA | 1825 |
|  | 2632R | CCTGGATATGTCGATGC |  |  |
| *Air-opnGq4* | 66F | CGTGCCCAACTTCATCATC | cDNA | 1609 |
|  | 97R | CAAAAGTGTGCGTTCTTCTG |  |  |
| *Air-opnGq4* | 66F | CGTGCCCAACTTCATCATC | gDNA | 1609 |
|  | 97R | CAAAAGTGTGCGTTCTTCTG |  |  |
| Intergenic region | PR-4-Fw-Air-OPNGq3 | TAATATAGCCTTGCCTGCCTCACTTGCC | cDNA | 1752 |
|  | PR-5-Rv_Air-OPNGq4 | GGAACAATCTCTGTCACAGATTCCTCGG |  |  |
| Intergenic region | PR-4-Fw-Air-OPNGq3 | TAATATAGCCTTGCCTGCCTCACTTGCC | gDNA | 1752 |
|  | PR-5-Rv_Air-OPNGq4 | GGAACAATCTCTGTCACAGATTCCTCGG |  |  |
