# Supplementary material for: Structural differences and differential expression among rhabdomeric opsins reveal functional change after gene duplication in the bay scallop, Argopecten irradians (Pectinidae)
Source: BMC Evol Biol. 2016 Nov 17;16:250. doi: 10.1186/s12862-016-0823-9 (PMC5114761; doi:10.1186/s12862-016-0823-9)
Supplement: Supplementary file 2 — Primers used to amplify scallop Gq-opsins and intergenic region between Air-opnGq3 and Air-opnGq4. (DOCX 13 kb) [file 12862_2016_823_MOESM1_ESM.docx]

**Additional file 1: Table S2.** G_q_-opsin sequences included in the phylogenetic analysis.

Asterisks represent sequences obtained through Porter et al. (2012). For additional information regarding sequence acquisition not available on Genbank, see supplementary material in Porter et al. (2012).

| **Identity** | **Phylum** | **Genus** | **species** | **common name** | **Genbank accession# or source** |
| --- | --- | --- | --- | --- | --- |
| Al.subulata | Mollusca | *Alloteuthis* | *subulata* | squid | Z49108 |
| Ap.californica1 | Mollusca | *Aplysia* | *californica* | sea hare | AASC01108363* |
| Ap.californica2 | Mollusca | *Aplysia* | *californica* | sea hare | AASC02005512* |
| Air-OPNGo1 | Mollusca | *Argopecten* | *irradians* | bay scallop | TBD |
| Air-OPNGq1 | Mollusca | *Argopecten* | *irradians* | bay scallop | KT426908 |
| Air-OPSGq2 | Mollusca | *Argopecten* | *irradians* | bay scallop | KT426909 |
| Air-OPNGq3 | Mollusca | *Argopecten* | *irradians* | bay scallop | KT426910 |
| Air-OPNGq4 | Mollusca | *Argopecten* | *irradians* | bay scallop | KT426911 |
| Bi.glabrata2 | Mollusca | *Biomphalaria* | *glabrata* | ram's horn snail | Genome, Dejong et al. 2004 |
| Bi.glabrata1 | Mollusca | *Biomphalaria* | *glabrata* | ram's horn snail | Genome, Dejong et al. 2004 |
| Cgi-OPNGq2B | Mollusca | *Crassostrea* | *gigas* | pacific oyster | Genome, Zhang et al. 2012 |
| Cgi-OPNGq1 | Mollusca | *Crassostrea* | *gigas* | pacific oyster | Genome, Zhang et al. 2012 |
| Cgi-OPNGq2A | Mollusca | *Crassostrea* | *gigas* | pacific oyster | Genome, Zhang et al. 2012 |
| En.dolfeini | Mollusca | *Enteroctopus* | *dofleini* | octopus | CAA30644.1 |
| Eu.scolopes | Mollusca | *Euprymna* | *scolopes* | ceph | ACB05673.1 |
| Lo.forbesi | Mollusca | *Loligo* | *forbesi* | squid | CAA40108.1 |
| Lo.pealei | Mollusca | *Loligo* | *pealei* | squid | AY450853 |
| L.gigantea1 | Mollusca | *Lottia* | *gigantea* | limpet | FC774055 |
| L.gigantea2 | Mollusca | *Lottia* | *gigantea* | limpet | Genome* |
| Mye-OPNGq1 | Mollusca | *Mizuhopecten* | *yessoensis* | scallop | AB006454 |
| Pi.fucata1 | Mollusca | *Pinctata* | *fucata* | pearl oyster | Genome, Takeuchi et al 2012 |
| Pi.fucata2 | Mollusca | *Pinctata* | *fucata* | pearl oyster | Genome, Takeuchi et al 2012 |
| Pma-OPNGq2 | Mollusca | *Placopecten* | *magellanicus* | sea scallop | Pairett & Serb 2013 |
| Pma-OPNGq3 | Mollusca | *Placopecten* | *magellanicus* | sea scallop | Pairett & Serb 2013 |
| Se.officinalis | Mollusca | *Sepia* | *officinalis* | cuttlefish | AF000947 |
| Tpa-OPNGq1 | Mollusca | *Todarodes* | *pacificus* | squid | X70498 |
| Ca.capitata | Annelida | *Capitella* | *capitata* | polychaete worm | Genome* |
| He.robusta1 | Annelida | *Helobdella* | *robusta* | leech | Genome* |
| He.robusta2 | Annelida | *Helobdella* | *robusta* | leech | Genome, scaffold_391* |
| P.dumerilii | Annelida | *Platynereis* | *dumerilii* | ragworm | AJ316544 |
| An.gambiae | Arthropoda | *Anopheles* | *gambiae* | mosquito | CAA76825.1 |
| An.gambiaeUV5 | Arthropoda | *Anopheles* | *gambiae* | mosquito | XP_001688790 |
| An.gambiaeUV7 | Arthropoda | *Anopheles* | *gambiae* | mosquito | XP_308329 |
| An.gambiaeUVB | Arthropoda | *Anopheles* | *gambiae* | mosquito | XP_319247.1 |
| Ap.melliferaA | Arthropoda | *Apis* | *mellifera* | bee | NM_001077825 |
| Ap.melliferaB | Arthropoda | *Apis* | *mellifera* | bee | U26026 |
| Ap.melliferaUV5 | Arthropoda | *Apis* | *mellifera* | bee | AAC13418 |
| Ap.melliferaUVB | Arthropoda | *Apis* | *mellifera* | bee | AF004168 |
| B.anynana | Arthropoda | *Bicyclus* | *anynana* | butterfly | 157502893 |
| B.anynanaUV | Arthropoda | *Bicyclus* | *anynana* | butterfly | AAL91507.1 |
| B.anynanaUVB | Arthropoda | *Bicyclus* | *anynana* | butterfly | AAY16527.1 |
| D.pulexBCR | Arthropoda | *Daphnia* | *pulex* | water flea | GL732562.1 |
| D.pulex | Arthropoda | *Daphnia* | *pulex* | water flea | Genome* |
| D.pulexUV5a | Arthropoda | *Daphnia* | *pulex* | water flea | EFX75461 |
| D.pulexUV5b | Arthropoda | *Daphnia* | *pulex* | water flea | EFX81332 |
| Dr.melanogast1 | Arthropoda | *Drosophila* | *melanogaster* | fruitfly | NP_524407.1 |
| Dr.melanogast2 | Arthropoda | *Drosophila* | *melanogaster* | fruitfly | AAA28734.1 |
| Dr.melanogast6 | Arthropoda | *Drosophila* | *melanogaster* | fruitfly | CAB06821.1 |
| Dr.melanogastUV3 | Arthropoda | *Drosophila* | *melanogaster* | fruitfly | AAA28854.1 |
| Dr.melanogastUV4 | Arthropoda | *Drosophila* | *melanogaster* | fruitfly | NP_476701.1 |
| Dr.melanogastUV5 | Arthropoda | *Drosophila* | *melanogaster* | fruitfly | AAC47426.1 |
| Dr.melanogastUV7 | Arthropoda | *Drosophila* | *melanogaster* | fruitfly | NP_524035 |
| Ha.adansoni1 | Arthropoda | *Hasarius* | *adansoni* | jumping spider | BAG14330.1 |
| Ha.adansoni2 | Arthropoda | *Hasarius* | *adansoni* | jumping spider | BAG14331.1 |
| Ha.adansoniUV5 | Arthropoda | *Hasarius* | *adansoni* | jumping spider | BAG14332.1 |
| Ix.scapularis | Arthropoda | *Ixodes* | *scapularis* | tick | XM_002408275.1 |
| Ix.scapularisUV7 | Arthropoda | *Ixodes* | *scapularis* | tick | Genome* |
| Li.polyphemusBCR | Arthropoda | *Limulus* | *polyphemus* | horseshoe crab | ACO05013 |
| Li.polyphemus | Arthropoda | *Limulus* | *polyphemus* | horseshoe crab | AAA02498.1 |
| Ne.oerstedii2 | Arthropoda | *Neogonodactylus* | *oerstedii* | stomatopod | DQ646870 |
| Ne.oerstedii3 | Arthropoda | *Neogonodactylus* | *oerstedii* | stomatopod | DQ646871 |
| Ne.oerstedii1 | Arthropoda | *Neogonodactylus* | *oerstedii* | stomatopod | DQ646869 |
| Pl.paykulli1 | Arthropoda | *Plexippus* | *paykulli* | jumping spider | BAG14333.1 |
| Pl.paykulli2 | Arthropoda | *Plexippus* | *paykulli* | jumping spider | BAG14334.1 |
| Pl.paykulliUV5 | Arthropoda | *Plexippus* | *paykulli* | jumping spider | BAG14335 |
| Tr.castaneum | Arthropoda | *Tribolium* | *castaneum* | flour beetle | ABA00706.1 |
| Tr.castaneumUV5 | Arthropoda | *Tribolium* | *castaneum* | flour beetle | ABW06837.1 |
| Br.belcheri6 | Chordata | *Branchiostoma* | *belcheri* | amphioxus | AB050611 |
| Br.floridae6 | Chordata | *Branchiostoma* | *floridae* | amphioxus | XP_002586119.1 |
| Br.belcheri | Chordata | *Branchiostoma* | *belcheri* | amphioxus | AB205400 |
| Br.floridae | Chordata | *Branchiostoma* | *floridae* | amphioxus | Genome* |
| Ci.intestinalis | Chordata | *Ciona* | *intestinalis* | tunicate | AABS01000008.1 |
| Ci.savignyi | Chordata | *Ciona* | *savignyi* | tunicate | Genome* |
| Da.rerio1A | Chordata | *Danio* | *rerio* | zebrafish | Genome* |
| Da.rerio1B | Chordata | *Danio* | *rerio* | zebrafish | Genome* |
| Da.rerio2 | Chordata | *Danio* | *rerio* | zebrafish | Genome* |
| Da.rerioGt.MWS | Chordata | *Danio* | *rerio* | zebrafish | NP_571250.1 |
| Da.rerioGt.PAR | Chordata | *Danio* | *rerio* | zebrafish | XP_003201482 |
| Da.rerioGt.PPIN | Chordata | *Danio* | *rerio* | zebrafish | NP_001005312.1 |
| Da.rerioGt.Rho1 | Chordata | *Danio* | *rerio* | zebrafish | BC164171.1 |
| Da.rerioGt.Rho2 | Chordata | *Danio* | *rerio* | zebrafish | NM_131254 |
| Da.rerioGt.SWS | Chordata | *Danio* | *rerio* | zebrafish | NP_571394.1 |
| Da.rerioGt.TMT | Chordata | *Danio* | *rerio* | zebrafish | NP_001112371.1 |
| Da.rerioGt.VA | Chordata | *Danio* | *rerio* | zebrafish | NM_131586 |
| Ga.gallus1 | Chordata | *Gallus* | *gallus* | chicken | NP_001038118.1 |
| Ga.gallus2 | Chordata | *Gallus* | *gallus* | chicken | AY882944 |
| Mu.musculus | Chordata | *Mus* | *musculus* | mouse | AF147789 |
| Xe.laevis1 | Chordata | *Xenopus* | *laevis* | frog | ABD37674.1 |
| Xe.laevis2 | Chordata | *Xenopus* | *laevis* | frog | Genome* |
| St.purpuratus | Echinodermata | *Strongylocentrotus* | *purpuratus* | sea urchin | XR_026330* |
| Du.japonica | Platyhelminthes | *Dugesia* | *japonica* | flatworm | CAD13146 |
| Gi.tigrina | Platyhelminthes | *Girardia* | *tigrina* | flatworm | CAB89516 |
| Sc.mansoni1 | Platyhelminthes | *Schistosoma* | *mansoni* | trematode worm | AF155134 |
| Sc.mansoni2 | Platyhelminthes | *Schistosoma* | *mansoni* | trematode worm | CD096414 |
| Sc.mansoni3 | Platyhelminthes | *Schistosoma* | *mansoni* | trematode worm | Smp_180030 |
| Sc.meditrranea | Platyhelminthes | *Schmidtea* | *mediterranea* | planaria | AF112361 |
