# Supplementary material for: Structural differences and differential expression among rhabdomeric opsins reveal functional change after gene duplication in the bay scallop, Argopecten irradians (Pectinidae)
Source: BMC Evol Biol. 2016 Nov 17;16:250. doi: 10.1186/s12862-016-0823-9 (PMC5114761; doi:10.1186/s12862-016-0823-9)
Supplement: Supplementary file 3 — Ramachandran plot values and C-scores for top Gq-opsin models. For each Air-OPNGq, the top five models reported by I-TASSER were analyzed for their quality using PROCHECK and the C-score. All the reported models have > 90% of their residues in allowed regions of the Ramachandran plot, indicating a good quality model. The C-scores for the best models was in the range of −3 to −2. While these values are lower than the suggested cutoff of −1.5, this is not unexpected for GPCRs because there are relatively few solved GPCR protein structures and GPCRs often show high sequence diversity. The best model for each Air-OPNGq (highlighted) was selected as the structure having the highest C-score and highest percentage of residues in allowed regions of the Ramachandran plot. (DOCX 14 kb) [file 12862_2016_823_MOESM5_ESM.docx]

**Additional file 5: Figure S2 Maximum likelihood phylogram of G_q_-opsins.** The phylogenetic tree is based on 96 aligned amino acid sequences with scallop *Argopecten irradians* G_o_-opsin as the outgroup. Support values (>50%) of nodes were generated by 1000 bootstrap replicates in RAxML. A black bar indicates the monophyletic G_q_-opsin clade.

**
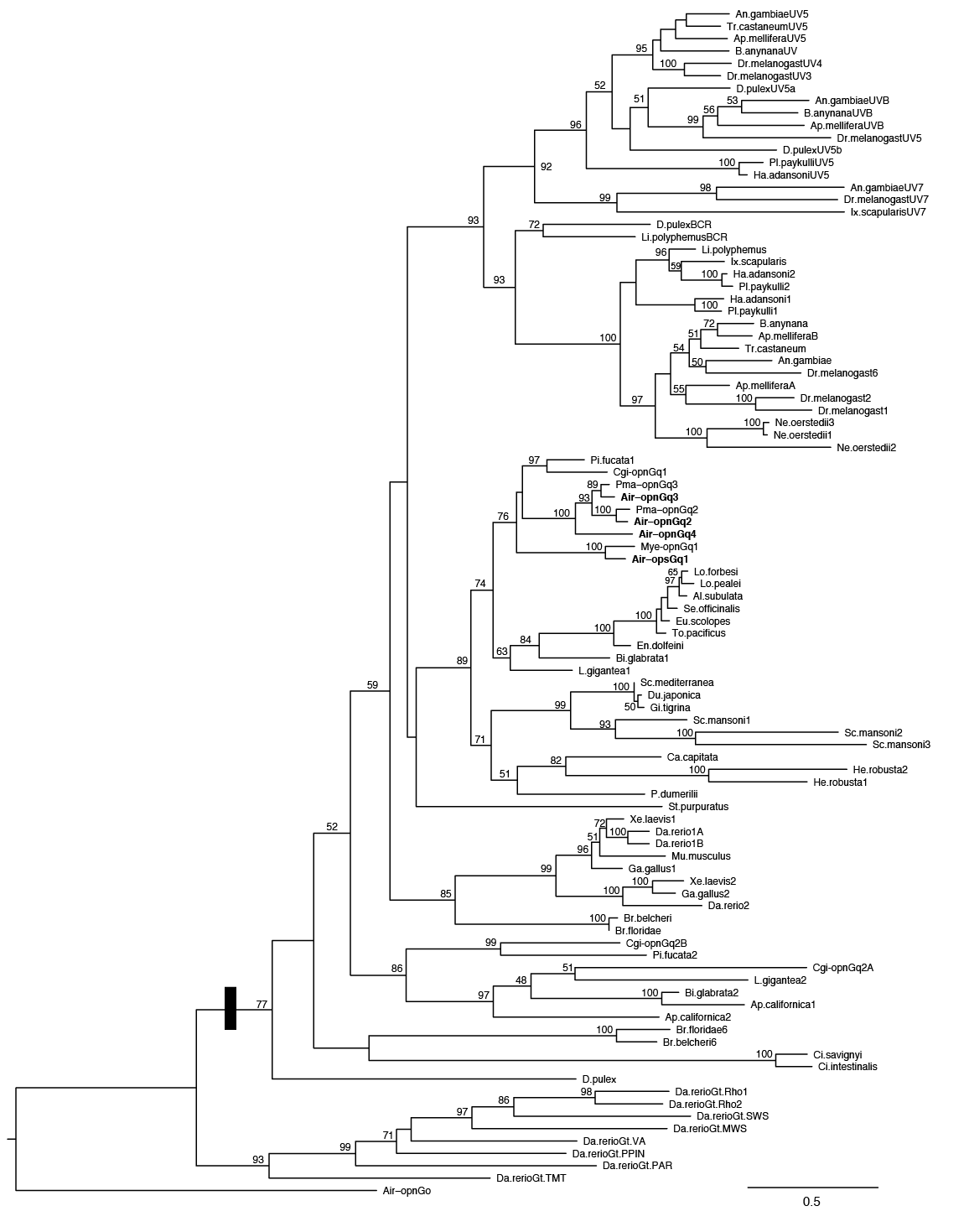
**
