# Supplementary material for: Structural differences and differential expression among rhabdomeric opsins reveal functional change after gene duplication in the bay scallop, Argopecten irradians (Pectinidae)
Source: BMC Evol Biol. 2016 Nov 17;16:250. doi: 10.1186/s12862-016-0823-9 (PMC5114761; doi:10.1186/s12862-016-0823-9)
Supplement: Supplementary file 4 — Bayesian inference phylogram of Gq-opsins. The phylogenetic tree is based on 96 aligned amino acid sequences with scallop Argopecten irradians Go-opsin as the outgroup. Support values at nodes are posterior probabilities >0.50. The grey box highlights a clade of bivalve opnGq1 not recovered in the ML analysis. A black bar indicates the monophyletic Gq-opsin clade. (DOCX 94 kb) [file 12862_2016_823_MOESM3_ESM.docx]

**Additional file 3: Table S3. Ramachandran plot values and C-scores for top G_q_-opsin models**

|  | **Model number** | **% residues in most favored regions** | **% residues in additionally allowed regions** | **% residues in generously allowed regions** | **% residues in disallowed regions** | **C-scores** |
| --- | --- | --- | --- | --- | --- | --- |
| **Air-OPNGq1** | **1** | **85.4** | **10.9** | **2.5** | **1.2** | **-1.95** |
|  | 2 | 87.3 | 8.6 | 2.3 | 1.9 | -2.05 |
|  | 3 | 83.6 | 12.7 | 2.1 | 1.6 | -2.53 |
|  | 4 | 84.7 | 10.6 | 3.7 | 0.9 | -3.06 |
|  | 5 | 85.4 | 10.2 | 2.1 | 2.3 | -2.53 |
|  |  |  |  |  |  |  |
| **Air-OPNGq2** | **1** | **85.6** | **10.6** | **1.8** | **2.0** | **-0.85** |
|  | 2 | 87.4 | 7.6 | 2.3 | 2.8 | -2.01 |
|  | 3 | 85.9 | 7.1 | 4.8 | 2.3 | -2.22 |
|  | 4 | 87.9 | 8.8 | 1.5 | 1.8 | -1.35 |
|  | 5 | 87.4 | 7.8 | 3.3 | 1.5 | -2.00 |
|  |  |  |  |  |  |  |
| **Air-OPNGq3** | **1** | **83.6** | **10.7** | **3.3** | **2.4** | **-2.17** |
|  | 2 | 86.0 | 9.2 | 2.6 | 2.2 | -2.33 |
|  | 3 | 88.8 | 8.3 | 1.5 | 1.3 | -2.44 |
|  | 4 | 84.0 | 11.6 | 3.5 | 0.9 | -2.56 |
|  | 5 | 86.0 | 7.9 | 3.5 | 2.6 | -2.70 |
|  |  |  |  |  |  |  |
| **Air-OPNGq4** | **1** | **87.2** | **8.5** | **2.4** | **1.9** | **-1.98** |
|  | 2 | 86.7 | 7.8 | 3.1 | 2.4 | -2.25 |
|  | 3 | 84.4 | 12.6 | 2.6 | 0.5 | -2.42 |
|  | 4 | 83.9 | 11.4 | 2.1 | 2.6 | -2.37 |
|  | 5 | 88.2 | 6.9 | 4.0 | 0.9 | -2.34 |
|  |  |  |  |  |  |  |
| **Tpa-OPNGq1** |  | 70.4 | 27.1 | 2.1 | 0.3 |  |

Note – For each Air-OPNGq, the top five models reported by I-TASSER were analyzed for their quality using PROCHECK and the C-score. All the reported models have > 90% of their residues in allowed regions of the Ramachandran plot, indicating a good quality model. The C-scores for the best models was in the range of -3 to -2. While these values are lower than the suggested cutoff of -1.5, this is not unexpected for GPCRs because there are relatively few solved GPCR protein structures and GPCRs often show high sequence diversity. The best model for each Air-OPNGq (highlighted) was selected as the structure having the highest C-score and highest percentage of residues in allowed regions of the Ramachandran plot.
