# Supplementary material for: Structural differences and differential expression among rhabdomeric opsins reveal functional change after gene duplication in the bay scallop, Argopecten irradians (Pectinidae)
Source: BMC Evol Biol. 2016 Nov 17;16:250. doi: 10.1186/s12862-016-0823-9 (PMC5114761; doi:10.1186/s12862-016-0823-9)
Supplement: Supplementary file 5 — Maximum likelihood phylogram of Gq-opsins. The phylogenetic tree is based on 96 aligned amino acid sequences with scallop, Argopecten irradians, Go-opsin as the outgroup. Support values (>50%) of nodes were generated by 1000 bootstrap replicates in RAxML. A black bar indicates the Gq-opsin clade. (DOCX 98 kb) [file 12862_2016_823_MOESM4_ESM.docx]

**Additional file 4: Figure S1. Bayesian inference phylogram of G_q_-opsins.** The phylogenetic tree is based on 96 aligned amino acid sequences with scallop *Argopecten irradians* G_o_-opsin as the outgroup. Support values at nodes are posterior probabilities >0.50. The grey box highlights a clade of bivalve *opnGq1* not recovered in the ML analysis. A black bar indicates the monophyletic G_q_-opsin clade.

**
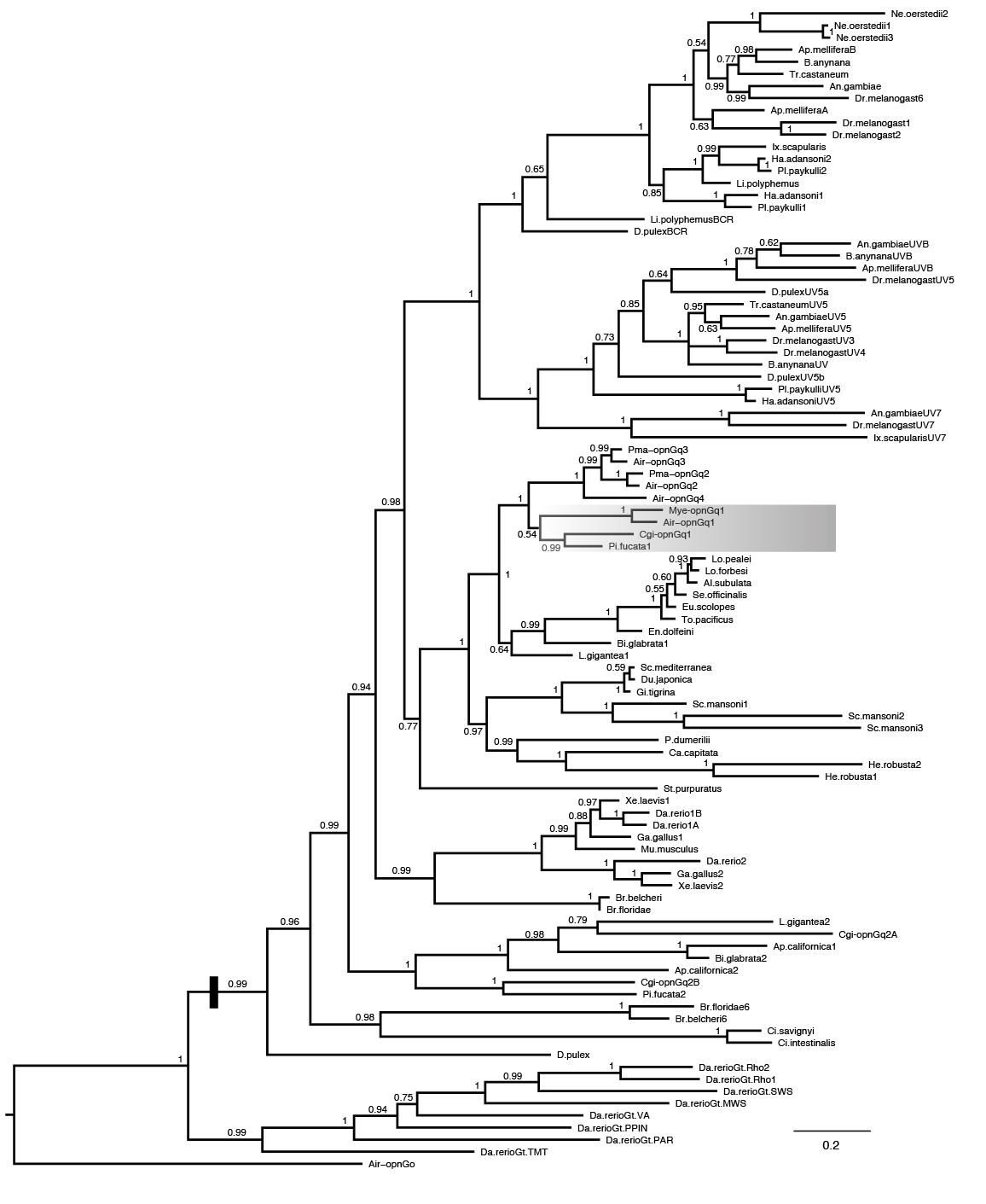
**
