# Supplementary material for: Structural differences and differential expression among rhabdomeric opsins reveal functional change after gene duplication in the bay scallop, Argopecten irradians (Pectinidae)
Source: BMC Evol Biol. 2016 Nov 17;16:250. doi: 10.1186/s12862-016-0823-9 (PMC5114761; doi:10.1186/s12862-016-0823-9)
Supplement: Supplementary file 6 — Fifty base pair alignment of 5′- and 3′-UTRs from the four scallop Gq-opsins. Vertical lines represent the beginning and end of the coding region. (DOCX 49 kb) [file 12862_2016_823_MOESM6_ESM.docx]

**Additional file 6: Figure S3.** Fifty base pair alignment of 5’- and 3’-UTRs from the four scallop G_q_-opsins. Vertical lines represent the beginning and end of the coding region.

5’ UTR


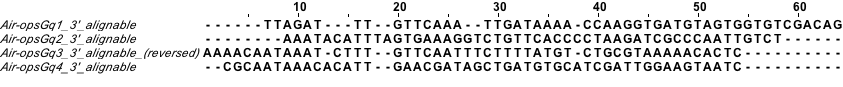

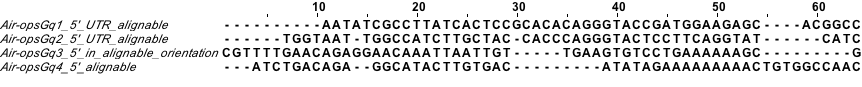


**Air-opnGq1**

**Air-opnGq2**

**Air-opnGq3**

**Air-opnGq4**

**M………..**

**M………..**

**M………..**

**M………..**

**Air-opnGq1**

**Air-opnGq2**

**Air-opnGq3**

**Air-opnGq4**

**………..**

**………..**

**………..**

**………..**

*

*

*

*

3’ UTR
